# PPR-DYW Protein EMP17 Is Required for Mitochondrial RNA Editing, Complex III Biogenesis, and Seed Development in Maize

**DOI:** 10.3389/fpls.2021.693272

**Published:** 2021-07-28

**Authors:** Yong Wang, Xin-Yuan Liu, Zi-Qin Huang, Yan-Yan Li, Yan-Zhuo Yang, Aqib Sayyed, Feng Sun, Zhi-Qun Gu, Xiaomin Wang, Bao-Cai Tan

**Affiliations:** ^1^Key Laboratory of Plant Development and Environmental Adaptation Biology, Ministry of Education, School of Life Sciences, Shandong University, Qingdao, China; ^2^Key Laboratory of Cell Activities and Stress Adaptations, Ministry of Education, School of Life Sciences, Lanzhou University, Lanzhou, China

**Keywords:** CcmF_C_, EMP17, pentatricopeptide repeat protein, mitochondrion, seed development, maize

## Abstract

The conversion of cytidines to uridines (C-to-U) at specific sites in mitochondrial and plastid transcripts is a post-transcriptional processing event that is important to the expression of organellar genes. Pentatricopeptide repeat (PPR) proteins are involved in this process. In this study, we report the function of a previously uncharacterized PPR-DYW protein, Empty pericarp17 (EMP17), in the C-to-U editing and kernel development in maize. EMP17 is targeted to mitochondria. The loss-function of EMP17 arrests maize kernel development, abolishes the editing at *ccmF*_*C*_-799 and *nad2*-677 sites, and reduces the editing at *ccmF*_*C*_-906 and -966 sites. The absence of editing causes amino acid residue changes in CcmF_C_-267 (Ser to Pro) and Nad2-226 (Phe to Ser), respectively. As CcmF_C_ functions in cytochrome *c* (Cyt*c*) maturation, the amount of Cyt*c* and Cyt*c*_1_ protein is drastically reduced in *emp17*, suggesting that the CcmF_C_-267 (Ser to Pro) change impairs the CcmF_C_ function. As a result, the assembly of complex III is strikingly decreased in *emp17*. In contrast, the assembly of complex I appears less affected, suggesting that the Nad2-226 (Phe to Ser) change may have less impact on Nad2 function. Together, these results indicate that EMP17 is required for the C-to-U editing at several sites in mitochondrial transcripts, complex III biogenesis, and seed development in maize.

## Introduction

The mitochondrion is a semi-autonomous organelle that provides energy and metabolites for cell activity. Plant mitochondrial genome inherits ∼5% genes from its prokaryotic ancestor, which encodes proteins of the respiratory complexes and ribosome, transfer RNAs (tRNAs), and ribosomal RNAs (rRNAs) (Clifton et al., 2004). Post-transcriptional processing of mitochondrial transcripts—including conversion of cytidines to uridines (C-to-U) editing, 5′- and 3′-terminus maturation, and intron splicing—is important for the function of the encoded proteins (Giege and Brennicke, 2001; Hammani and Giege, 2014). C-to-U editing is a major event of post-transcriptional processing, as it often restores the conserved amino acids (Kotera et al., 2005; Liu et al., 2013), generates translation start or stop codon (Kadowaki et al., 1995), regulates intron splicing (Xu et al., 2020), and enhances tRNA precursor efficient processing (Fey et al., 2002). In flowering plants, C-to-U editing occurs predominantly in mitochondrial transcripts with 300–600 editing sites (Notsu et al., 2002; Handa, 2003; Mower and Palmer, 2006; Bentolila et al., 2013; Wang et al., 2019), in comparison to the 20–40 sites in plastid transcripts (Tsudzuki et al., 2001; Tillich et al., 2005). A lack of editing often causes a deleterious impact on plant growth and development, and in some cases, even embryo lethality (Li et al., 2014; Xie et al., 2016; Wang et al., 2017; Xiao et al., 2018).

Although many factors have been identified to function in the C-to-U editing, the exact mechanism remains to be deciphered (Sun et al., 2016; Small et al., 2020). Pentatricopeptide repeat (PPR) proteins have been identified to play an essential role in editing site recognition. PPR proteins belong to one of the largest protein families in plants, with over 400 members in terrestrial plants (Lurin et al., 2004; O’Toole et al., 2008; Fujii and Small, 2011; Wei and Han, 2016). PPRs are classified into two classes: P-class and PLS-class (Small and Peeters, 2000; Lurin et al., 2004; Cheng et al., 2016). The P-class PPR proteins consist of *bona fide* P-motifs with 35 amino acids, while the PLS-class proteins harbor P-, L- (35–36 amino acids), and S-motifs (31 amino acids), and often carry an E, E+, and/or DYW domain at the C-terminus. As ubiquitous RNA binding factors, PPR proteins were found to take part in almost all of the post-transcriptional processing in mitochondria and plastids (Barkan and Small, 2014). The PLS-class PPR proteins mediate the C-to-U editing by specific binding to the nucleotide sequence upstream of the editing site through the PPR motifs (Tasaki et al., 2010; Okuda and Shikanai, 2012; Jiang et al., 2018). Furthermore, C-to-U editing also involves deamination of the cytidine (Blanc et al., 1995; Yu and Schuster, 1995), and the DYW domain possesses the cytidine deaminase (CDA) activity as demonstrated in PpPPR65 and PpPPR56 (Oldenkott et al., 2019; Hayes and Santibanez, 2020). The DYW domain also contains the motif of zinc-binding signature residues [HxE(x)nCxxC] commonly found in deaminases (Bhattacharya et al., 1994). Most DYW domains in higher plant PPR-DYW proteins contain the CDAs-like signature motif, which has been found to be indispensable for RNA editing (Boussardon et al., 2014; Hayes et al., 2015; Wagoner et al., 2015).

Accumulating evidence suggests that C-to-U editing is carried out by a protein complex, termed “editosome” (Sun et al., 2016; Small et al., 2020). Recent studies have shown that proteins of distinct families are involved in RNA editing in addition to the PPRs. These include multiple organelle RNA editing factors (MORFs)/RNA-editing factor interacting proteins (RIPs), organelle RRM proteins (ORRMs), organelle zinc-finger 1 (OZ1), and other proteins as reviewed by Small et al. (2020). MORFs/RIPs harboring a conserved MORF/RIP motif have been shown to be required for the editing at a large number of sites in mitochondria and nearly all sites in plastids in *Arabidopsis* (Bentolila et al., 2013). MORFs also selectively interact with PPR proteins and form homo- or heterodimers (Bentolila et al., 2012; Takenaka et al., 2012; Glass et al., 2015; Zehrmann et al., 2015; Haag et al., 2017). ORRM proteins, on the other hand, are required for the editing at many sites in mitochondria (ORRM2, ORRM3, ORRM4, and ORRM5) (Shi et al., 2015, 2016, 2017), and more than half of the sites in plastids in *Arabidopsis* (ORRM1 and ORRM6) (Sun et al., 2013; Hackett et al., 2017). ORRM proteins can interact with themselves, other ORRMs, and MORFs (Sun et al., 2013; Shi et al., 2015, 2016). ORRM1, specifically, is co-purified with OZ1, which is required for the editing at 81% plastid target Cs (Sun T. et al., 2015). A recent report showed that an active editing complex contains PPRs, RIPs, ORRM1, OZ1, and ISE2 in maize chloroplasts (Sandoval et al., 2019).

Plant mitochondria harbor two mono-hemic *c*-type cytochromes: Cytochrome *c* (Cyt*c*) and Cytochrome *c1* (Cyt*c*_1_). They are the essential factors of the oxidative phosphorylation (OXPHOS) chain. Cyt*c*_1_, anchored in the inner membrane, is a core subunit of complex III. On the other hand, Cyt*c*, peripherally associated with the inner membrane, shuttles electrons from complex III to complex IV (Giege et al., 2008). After translation, apo-cytochrome *c* peptides are no longer functional. They have to undergo a maturation process in which the heme prosthetic groups are covalently attached to apo-Cyt*c*_1_ and apo-Cyt*c via* thioether bonds. In gram-negative bacteria, 8–9-ccm genes (*ccmA* to *ccmI*) are involved in the cytochrome *c* maturation (CCM), referred to as the CCM pathway or system I (Thony-Meyer et al., 1995). Plant mitochondria inherit the CCM pathway from the prokaryote ancestor (Giege et al., 2008), and in maize specifically, the CCM pathway involves seven proteins: three (CCMA, CCME, and CCMH) encoded by the nuclear genes and four (CcmB, CcmC, CcmF_N_, and CcmF_C_) by the mitochondrial genes (Clifton et al., 2004; Meyer et al., 2005; Rayapuram et al., 2008). Furthermore, loss-of-function of CCM factors often causes embryo lethality in plants. For example, a deficiency of CcmF_N_ protein in the *emp7* and *ppr27* mutants results in a deficiency of Cyt*c* and Cyt*c*_1_, leading to impaired assembly of complex III and arrested seed development in maize (Sun F. et al., 2015; Liu et al., 2020).

In this study, we report the function of an uncharacterized mitochondrion-targeted PPR-DYW protein, erythropoietin-mimetic peptide 17 (EMP17), in maize. The results demonstrate that EMP17 is required for the editing at four sites of *ccmF*_*C*_ and *nad2* transcripts in mitochondria. Deficiency in the editing at *ccmF*_*C*_-799 and *nad2*-677 causes amino acid changes in the encoded protein. We provide further evidence that the Ser-to-Pro change at CcmF_C_-267 impairs the CcmF_C_ activity, blocks the maturation of Cyt*c* and Cyt*c*_1_, and disrupts the assembly of complex III, which attributes to the arrest of seed development in the *emp17* maize mutant.

## Materials and Methods

### Plant Materials and Growth Conditions

The *emp17* mutant was isolated from the UniformMu mutagenic population in nearly isogenic W22 background (McCarty et al., 2005). The plants were grown in the experimental field of Shandong University in Qingdao, Shandong province under natural conditions.

### DNA Extraction and Linkage Analysis

Genomic DNA (gDNA) was extracted by using a urea-phenol-chloroform-based method as described by Tan et al. (2011). The Emp17-F1 primer and *Mu* specific primer TIR8 (TIR8a:TIR8b:TIR8c:TIR8d = 1:2:2:1) were used to detect the *Mu* insertion in *Emp17*, and the Emp17-F1/Emp17-R1 primer pair was used to amplify the wild type *Emp17*.

### Construction of *Emp17* Overexpression Plants

The protein-coding region of *Emp17* complementary DNA (cDNA) was placed downstream of the maize ubiquitin 1 (Ubi-1) promoter in the pUNTF vector. This pUNTF-Emp17 construct was transformed into maize inbred KN5585 *via* callus transformation. Positive transgenic plants of *Emp17* were screened by using the *Bar* gene, and further verified by PCR using the Ubi-F primer anchored to the ubiquitin sequence in the vector and the *Emp17* specific primer Emp17-R1.

### Subcellular Localization

The 420 bp, 5′-sequence of *Emp17* encoding the 140 amino acids of the N-terminus region was cloned into pENTR/D-TOPO vector (Invitrogen in ThermoFisher Scientific, http://www.thermofisher.com), and then transferred to the pBI221 vector to create the Emp17^*N*140^-GFP fusion by the Gateway site-specific recombination. The resulting construct, pBI221-Emp17^*N*140^-GFP, was introduced into *Arabidopsis* protoplasts. MitoTracker Red was used as a marker for mitochondria. The fluorescence signals were detected under a ZEISS LSM 880 confocal microscope.

### Light Microscopy of Cytological Sections

Wild type and *emp17* kernels were harvested at 10 and 14 days after pollination (DAP) from the selfed ears of *emp17* (+/−) heterozygous plants. The kernels were fixed in 4% paraformaldehyde at 4°C for 24 h. The fixed kernels were dehydrated, cleared, infiltrated, embedded, sectioned, stained, and observed as described by Liu et al. (2013).

### RNA Extraction, RT-PCR, and qRT-PCR

Total RNA was extracted from developing kernels and other tissues of maize with the RNeasy Plant Mini Kit (Qiagen, http://www.qiagen.com) according to the manufacturer’s instruction. The potential gDNA contamination was removed through Dnase I (New England Biolabs, www.neb.sg) treatment. The single-stranded cDNA was obtained by reverse transcription reaction using the Transcriptor First Strand cDNA Synthesis kit (Thermo Fisher Scientific, http://www.thermofisher.com). Quantitative real-time PCR (qRT-PCR) with SYBR green (Bio-Rad, http://www.bio-rad.com) was performed in a Roche Light Cycler 96. The relative transcript level was calculated as described in previous studies (Wang et al., 2019). *ZmActin* (GRMZM2G126010) was used as a control in RT-PCR and qRT-PCR. Detailed information of the primers was listed in Supplementary Table 1.

### Direct Sequencing of RT-PCR Amplicons

RNA sample preparation of the wild type and *emp17* kernels and reverse transcription were performed as described above. The resulting cDNA was used as a template to amplify the 35 mitochondrial predicted-protein coding genes by RT-PCR. RNA editing was analyzed by directly sequencing the RT-PCR amplicons. The primers were listed in Supplementary Table 1. Three biological repetitions were analyzed.

### Blue Native-PAGE and Mitochondrial Complexes Assembly and Activity Assay

Crude mitochondria were extracted from the wild type and *emp17* kernels at 14 DAP as described previously (Li et al., 2014). Mitochondrial complexes were separated by the blue native polyacrylamide gel electrophoresis (BN-PAGE) assay. In-gel staining of complex I and supercomplex I + III_2_ activity was performed as described previously (Meyer et al., 2009). The assembly of complex III and complex V was detected by transferring the protein complexes to nitrocellulose membranes and hybridized with anti-Cyt*c*_1_ and anti-Atp1 antibodies. In-gel staining of complex IV was detected as described by Wang et al. (2020). Crude mitochondria were extracted and protein concentration was determined by the Bradford assay kit (Bio-Rad, http://www.bio-rad.com). For Western detection of complex proteins, 8 μg protein from *emp17*, and 8 μg, 4 μg (1/2), and 2 μg (1/4) protein from wild type were subjected to sodium dodecyl sulfate polyacrylamide gel electrophoresis (SDS-PAGE). Proteins were transferred to nitrocellulose membranes and detected by anti-Nad9, anti-Cyt*c*_1_, anti-Cyt*c*, anti-Cox2, and anti-Atp1 as described previously (Sun F. et al., 2015).

### Respiration Rate Assay

The respiration rates were determined according to the protocol described by Wang et al. (2015). O_2_ consumption of the wild type and *emp17* kernels was measured at dark by using a Chlorolab II liquid-phase oxygen electrode (Hansatech, http://www.hansatech-instruments.com/) in a reaction buffer containing 10 mM HEPES, 10 mM MES, and 2 mM CaCl_2_ (where pH was adjusted to 6.8 with KOH). Total respiration rate (*V*_*t*_) was defined by the O_2_ consumption before adding any respiration inhibitor. The cytochrome pathway capacity (*V*_*cyt*_) was determined by *V*_*t*_ minus the O_2_ consumption in the presence of 2 mM salicylhydroxamic acid (SHAM) and the alternative pathway capacity (*V*_*alt*_) was defined by *V*_*t*_ minus the O_2_ consumption in the presence of 2 mM potassium cyanide (KCN). All three respiration rates were expressed as nmol O_2_ min^–1^ g^–1^ fresh weight.

## Results

### EMP17 Is a Mitochondrion-Targeted PPR-DYW Protein

**FIGURE 1:**
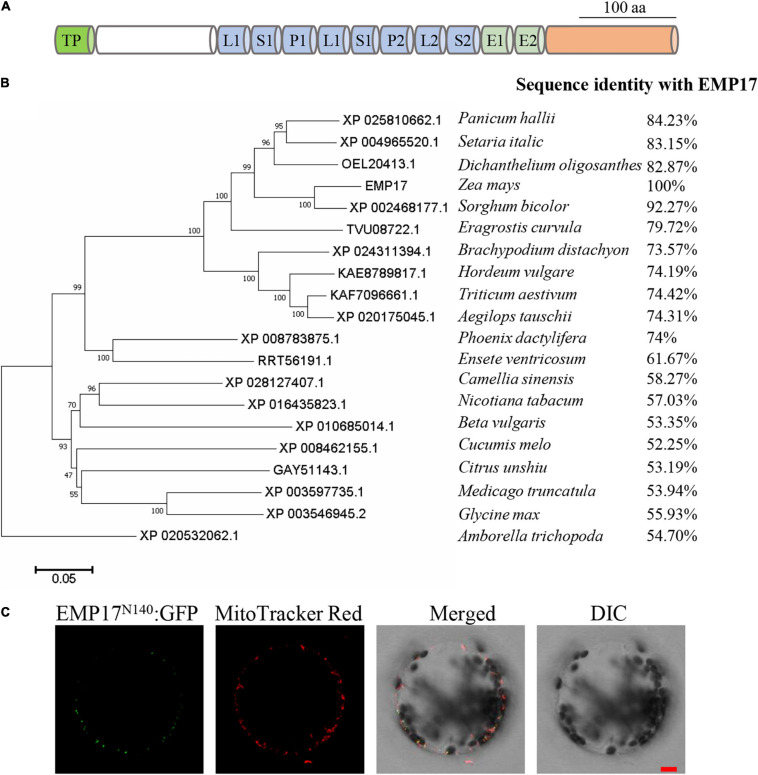
*Emp17* encodes a canonical PPR-DYW protein localized in mitochondria. **(A)** The protein structure of EMP17. aa, amino acid; TP, targeted peptide; L, PPR-like long motif; S, PPR-like short motif. **(B)** Evolutionary relationships of putative EMP17 orthologs. The evolutionary history was inferred using the Neighbor-Joining method. The percentage of replicate trees in which the associated taxa clustered together in the bootstrap test (1,000 replicates) are shown next to the branches. **(C)** The subcellular localization of EMP17. The 140 amino acids fragment from the N-terminus of EMP17 was fused with green fluorescence protein (EMP17^*N*140^:GFP) and transiently expressed in *Arabidopsis* protoplasts. Fluorescence signals were observed by confocal microscope ZEISS LSM 880. The mitochondria were stained by MitoTracker Red. DIC, differential interference contrast; Bar = 10 μm.

**FIGURE 2:**
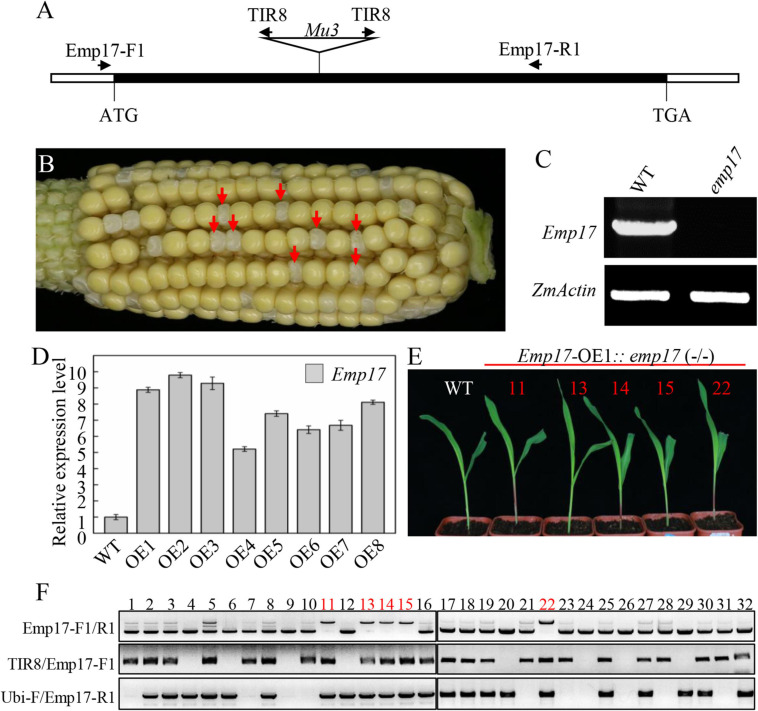
Mutation of *Emp17* arrests maize embryogenesis and endosperm development. **(A)** The gene structure of *Emp17*. The *Mu* transposon insertion was marked by a triangle. **(B)** A selfed ear of the *emp17* heterozygous plant. The empty pericarp kernels (*emp17*) are marked by red arrows. **(C)** RT-PCR analysis of *Emp17* transcription profiling in wild type and *emp17*. **(D)** The expression profiles of *Emp17* in wild type and *Emp17* overexpression plants. **(E)** The phenotype of *Emp17-OE1*:*emp17* (–/–) and wild type (WT) seedlings. **(F)** Genotype analysis of seedlings. The bands amplified by PCR using TIR8/Emp17-F1 primers indicate the *Mu* insertion in *Emp17*. The bands amplified by PCR using Ubi-F/Emp17-R1 primers indicate *Emp17* transgenic plants. The bands amplified by PCR using Emp17-F1/R1 primers indicate *Emp17* gene containing a *Mu* insertion (the longer bands) in the *Emp17-OE1*:*emp17* (–/–) seedlings, and the wild type *Emp17* gene (the shorter bands) in *emp17* (+/−) and WT, respectively.

PPR proteins form one of the largest protein families in maize with over 520 members annotated in the B73 genome. Among these, 82 are classified as the PPR-DYW subgroup proteins (Wei and Han, 2016). However, only six PPR-DYW proteins in maize have been fully characterized thus far: PPR2263 (Sosso et al., 2012), EMP5 (Liu et al., 2013), EMP18 (Li et al., 2019), EMP21 (Wang et al., 2019), PPR27 (Liu et al., 2020), and DEK46 (Xu et al., 2020). GRMZM2G019689 is a PPR-DYW subgroup protein encoded by an intron-less gene, hereafter referred to as “*Emp17*” (Figures 1A, 2A). EMP17 consists of 645 amino acid residues, and possesses 8 PPR motifs, an E1 and E2 domain, and a DYW domain with the conserved CDAs-like signature residues (HxE(x)nCxxC) (Figure 1A and Supplementary Figure 1). qRT-PCR analysis showed that *Emp17* was ubiquitously expressed in major tissues in maize, with a relatively high expression in stem and leaf (Supplementary Figure 2). Phylogenetic analysis revealed an extensive conservation in the protein sequences from *Amborella trichopoda* to mono- and eudicotyledonous species (Figure 1B). However, no clear orthologs were found in *Oryza sativa, Arabidopsis thaliana, Brassica napus*, and *Gossypium hirsutum*.

EMP17 was predicted to localize in mitochondria by the TargetP^1^ and Predotar algorithms.^2^ To experimentally localize EMP17, the N-terminal region containing 140 aa of EMP17 was fused with the green fluorescent protein (GFP) and then transiently expressed in *Arabidopsis* protoplasts. GFP signals were detected in punctated dots that merged with the mitochondria stained by the MitoTracker Red (Figure 1C). No GFP signals were found in other cellular compartments (Figure 1C), indicating that EMP17 is specifically localized in mitochondria.

### Loss of the EMP17 Function Severely Arrests Embryogenesis and Endosperm Development in Maize

To study the function of EMP17, a *Mutator* (*Mu*) insertion mutant (*emp17*) was isolated from the UniformMu mutagenic population (McCarty et al., 2005). A *Mu3* element was confirmed to be inserted at +660 bp from the translation start codon of *Emp17* (Figure 2A). The selfed progenies of *emp17* (+/−) heterozygous plants produced about 1/4 kernels with an empty pericarp phenotype (Figure 2B), and no wild type *Emp17* transcript was detected in the embryo and endosperm of the empty pericarp kernels (Figure 2C). Linkage analysis in an F2 population showed that only plants segregating emp mutant phenotype carried *Mu* insertion in *Emp17*, indicating that the *Mu* insertion in *Emp17* is either tightly linked to or the cause of the emp phenotype (Figure 2B and Supplementary Figure 3).

To confirm whether *Emp17* is the causal gene for the empty pericarp phenotype, we created transgenic plants over-expressing *Emp17* (*Emp17*-OE) in the inbred line KN5585 by placing *Emp17* under the Ubi-1 promoter. Eight independent transgenic lines (*Emp17*-OE1 to OE8) were obtained, and the expression level of *Emp17* in these lines was 5–9.5 times higher than that in the wild type as detected by qRT-PCR (Figure 2D). We crossed *Emp17*-OE1 with the *emp17* (+/−) heterozygous plants and selfed the F1 to obtain the F2 progeny. The F2 seedlings were genotyped by PCR. To distinguish between the endogenous *Emp17* and transgene *Emp17*, the Emp17-F1 primer was anchored to the 5’-UTR of the endogenous *Emp17*, ensuring that it could not anneal to the transgene that lacks this 5′-UTR sequence. The ubiquitin specific primer Ubi-F and *Mu* primer TIR8 were then used. Thus, the Emp17-F1/R1 primer pair amplified the endogenous *Emp17*, the Ubi-F/Emp17-R1 pair amplified the transgene, and the TIR8/Emp17-F1 pair detected the *Mu3* insertion in *Emp17*. Genotyping 32 of the F2 seedlings identified 5 seedlings that were homozygous for *emp17* harboring the *Emp17* transgene (Figures 2E,F). The Emp17-F1/R1 primer pair amplified a larger fragment in these five *emp17* (−/−) seedlings, which were proven to contain the *Mu3* element. These five seedlings showed normal growth and development compared with the wild type (Figure 2E), indicating over-expression of *Emp17* rescued the embryo-lethal phenotype of this mutant. These results demonstrate that *Emp17* (GRMZM2G019689) is the causal gene for the empty pericarp phenotype of maize kernels in *emp17*.

**FIGURE 3:**
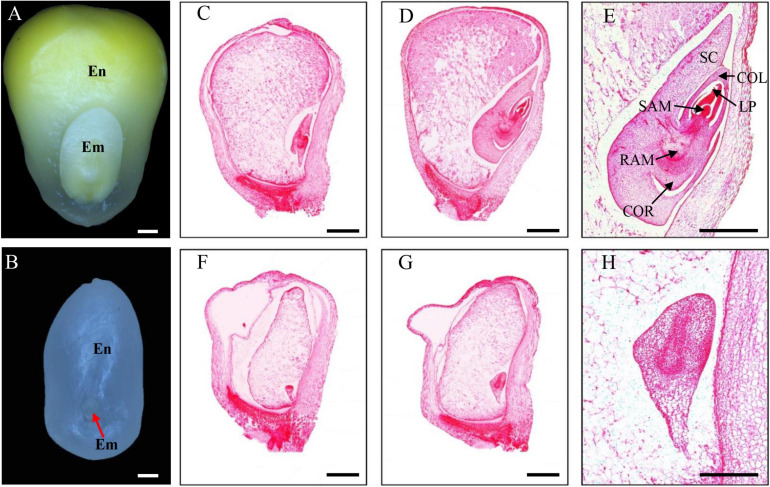
The *emp17* mutation arrests maize embryogenesis and endosperm development. **(A,B)** The embryo (Em) and endosperm (En) of the wild type **(A)** and *emp17* **(B)** at 14 days after pollination (DAP). **(C–H)** Paraffin section of WT **(C–E)** and *emp17* **(F–H)** developing kernels. **(C,F)** 10 DAP; **(D,E,G,H)** 14 DAP. SC, scutellum; COL, coleoptile; LP, leaf primordia; SAM, shoot apical meristem; RAM, root apical meristem; COR, coleorhiza. Bars = 1 mm in **(A–D,F,G)** and 500 μm in **(E,H)**.

The *emp17* kernels were substantially smaller than the wild type siblings throughout kernel development. At 14 DAP, the *emp17* embryo and endosperm was smaller than the wild type (Figures 3A,B). Paraffin sectioning indicated that the embryogenesis and endosperm development were severely arrested in *emp17*. At 10 DAP, leaf primordia (LP), shoot apical meristem (SAM), and root apical meristem (RAM) were clearly developed in the wild type embryo (Figure 3C). Conversely, the mutant embryo only proceeded to the early transition stage (Figure 3F). At 14 DAP, the wild-type embryo entered the late embryogenesis stage (Figures 3D,E), while the mutant embryo remained at the transition stage and the endosperm was arrested at the cellularization stage (Figures 3G,H).

### Loss-of-Function of EMP17 Abolishes the Editing at *ccmF*_*C*_-799 and *nad2*-677 Sites

**FIGURE 4:**
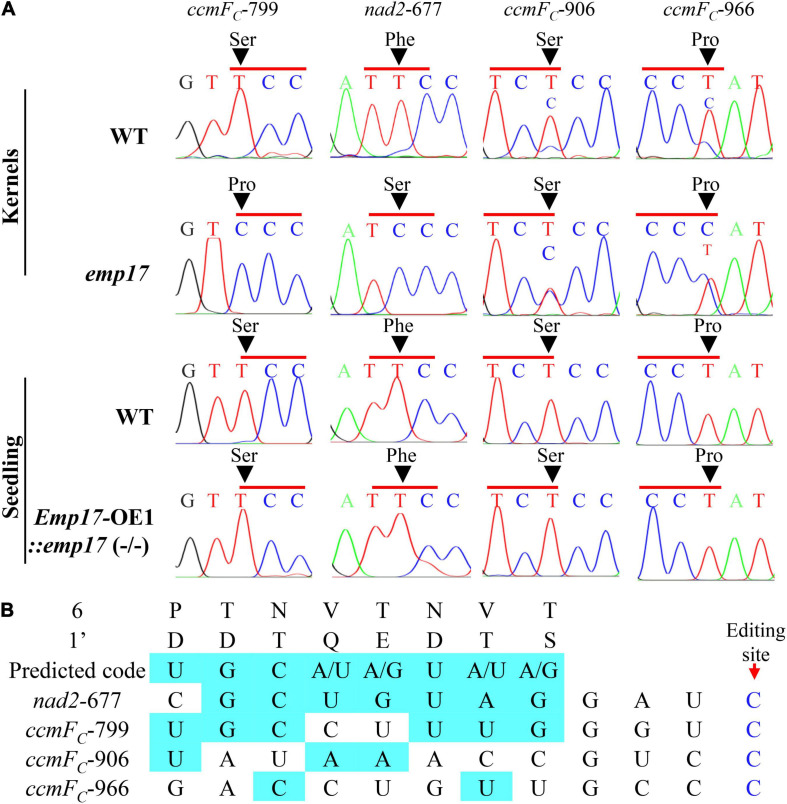
Loss of function of EMP17 abolishes the editing at *ccmF*_*C*_-799 and *nad2*-677 sites. **(A)** The defective editing sites in the *emp17* mutant. Sites subject to defective editing marked by arrows. **(B)** Alignment of the amino acid residues at position 6 and 1′ in each PPR motif of EMP17 with -4 to -11 bp upstream sequence of these four defective editing sites.

As previously reported, most of the known PPR-DYW proteins function in the C-to-U editing of organellar RNA (Guillaumot et al., 2017; Wang et al., 2019). The maize mitochondrial genome was predicted to encode 22 electron transport chain proteins, 11 ribosomal proteins, a maturase MatR, and a membrane transporter protein MttB (Clifton et al., 2004). These 35 gene transcripts were amplified from the *emp17* mutant and wild type kernels, and directly sequenced. Comparison of the sequences revealed that the C-to-U editing at *ccmF*_*C*_-799 and *nad2*-677 sites was abolished in *emp17*, and completely edited in the wild type (Figure 4A). In addition, the editing at *ccmF*_*C*_-906 and -966 sites was substantially decreased in *emp17* in comparison with the wild type (Figure 4A). In the *Emp17*-OE1:*emp17* (−/−) seedlings, the editing at *nad2*-677, *ccmF*_*C*_-799, -906, and *-*966 sites was restored (Figure 4A). These results indicate that the loss-of-function of *Emp17* abolishes the editing at *ccmF*_*C*_-799 and *nad2*-677, and decreases the editing at *ccmF*_*C*_-906 and -966.

PPR proteins recognize the target RNA sequence in a “one-PPR motif: one-nucleotide” manner, in which the sixth amino acid residue in the first PPR-motif and the 1′ amino acid residue in the next PPR-motif specifies the base of the RNA sequence (Barkan et al., 2012). Based on this code, the PPR motifs of EMP17 were aligned with the upstream sequence of *nad2*-677, *ccmF*_*C*_-799, -906, and -966 sites. The results showed that the codes aligned mostly with the *ccmF*_*C*_-799 and *nad2*-677 sites, but poorly with the *ccmF*_*C*_-906 and -966 sites (Figure 4B), suggesting that EMP17 probably binds strongly to the upstream sequences of the *ccmF*_*C*_-799 and *nad2*-677 sites, but weakly with the sequences of the *ccmF*_*C*_-906 and -966 sites.

### The Ser^267^ Residue in CcmF_C_ and Phe^226^ Residue in Nad2 Are Conserved in Plants

**FIGURE 5:**
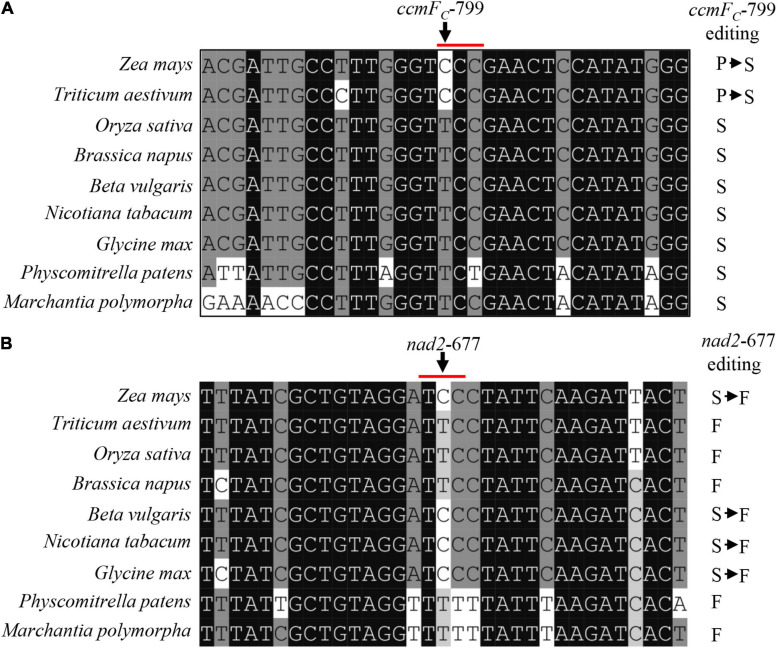
The amino acid residues encoded by *ccmF*_*C*_-799 and *nad2*-677 in multiple species. **(A,B)** Alignment of the neighboring gDNA sequences of *ccmF*_*C*_ and *nad2*. The gDNA and cDNA sequences were derived from GenBank/EST and GenBank/EMBL databases. The abolished editing sites in the *emp17* mutant are arrowed.

The deficient editing at *ccmF*_*C*_-799 and *nad2*-677 in the *emp17* mutant led to a Ser-to-Pro change at CcmF_C_-267 and a Phe-to-Ser change at Nad2-226, respectively (Figure 4A). Alignment of both gDNA and cDNA sequences of the *ccmF*_*C*_ and *nad2* orthologs indicated that these two amino acids were conserved in lower plants (*Physcomitrella patens* and *Marchantia polymorpha*), dicots (*Glycine max, Nicotiana tabacum, Beta vulgaris*, and *Brassica napus*), and monocots (*Zea mays, Triticum aestivum*, and *Oryza sativa*) (Figures 5A,B). The conservation of these two amino acid residues implies that these residues are probably important to the functional integrity of the CcmF_C_ and Nad2 proteins.

### Mitochondrial Complex III Biogenesis Is Severely Reduced in *emp17*

**FIGURE 6:**
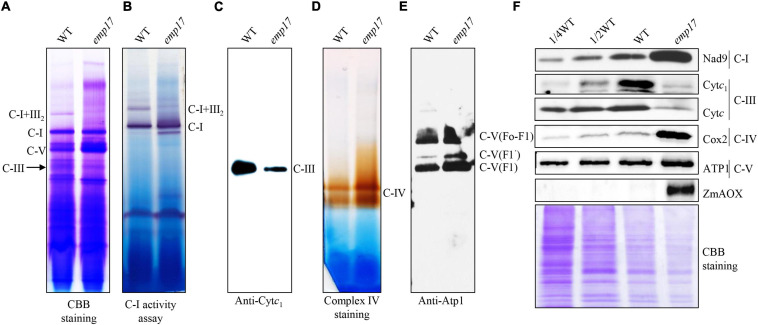
Mutation of *Emp17* compromises the assembly of the mitochondrial complex III and supercomplex I + III_2_. **(A)** Blue native-PAGE (BN-PAGE) analysis of the assembly of complex I and supercomplex I + III_2_. The gel was stained with Coomassie Brilliant Blue (CBB). **(B)** In-gel staining of the nicotinamide adenine dinucleotide (NADH) dehydrogenase activity of complex I and supercomplex I + III_2_. The loading control is Dihydrolipoamide dehydrogenase (DLDH) activity. **(C)** Western blotting assay based on antibodies against Cyt*c*_1_ (complex III). **(D)** In-gel activity staining of mitochondrial complex IV. **(E)** Western blotting assay based on antibody against Atp1 (complex V). **(F)** Western blotting assay with antibody against Nad9, Cyt*c*_1_, Cyt*c*, Cox2, Atp1, and ZmAOX. CBB staining was used for loading control. C-I: complex I, C-III: complex III, C-I + III_2_: supercomplex I + III_2_, C-V: complex V.

Nad2 is a subunit located in the hydrophobic arm of mitochondrial complex I (Braun et al., 2014), and CcmF_C_ is involved in the maturation of Cyt*c* and Cyt*c*_1_, which are components of mitochondrial complex III (Giege et al., 2008). To assess the impact of the amino acid residue alteration on these complexes, we analyzed the assembly and activity of mitochondrial complexes and the complex proteins in *emp17*. Mitochondria were isolated from the embryo and endosperm of the *emp17* mutant and wild type, respectively. The mitochondrial complexes were separated with BN-PAGE. Coomassie Brilliant Blue (CBB) staining showed that the level of complex I was comparable between *emp17* and wild type, but the levels of complex III and supercomplex I + III_2_ were remarkably decreased in *emp17* (Figure 6A). Furthermore, in-gel staining of the NADH dehydrogenase activity showed consistent results (Figure 6B). Western blot analysis indicated that the level of complex III as detected by anti-Cyt*c*_1_ antibody was drastically decreased in *emp17* (Figure 6C). Similarly, western blotting detection of complex V using anti-Atp1 antibody and in-gel staining of complex IV activity indicated that complex V and complex IV were increased in *emp17* as compared with wild type (Figures 6D,E). Western blot assays using anti-Nad9, Cyt*c*_1_, Cyt*c*, Cox2, and Atp1 antibodies showed that the levels of Cyt*c* and Cyt*c*_1_ was dramatically reduced. On the other hand, the level of Nad9 and Cox2 was substantially increased and the level of Atp1 was moderately increased in *emp17* as compared with wild type (Figure 6F). These results indicate that the abolished editing at *nad2*-677 site causing the Phe-to-Ser change at Nad2-226 appears not to significantly affect the assembly of mitochondrial complex I. However, the Ser-to-Pro change at CcmF_C_-267 severely inhibits the maturation of Cyt*c*_1_ and Cyt*c* and biogenesis of complex III, implying a critical role of Ser^267^ to the function of CcmF_C_.

### *Emp17* Loss of Function Decreases Cytochrome Respiration Rate and Increases *ZmAOX* Expression

**FIGURE 7:**
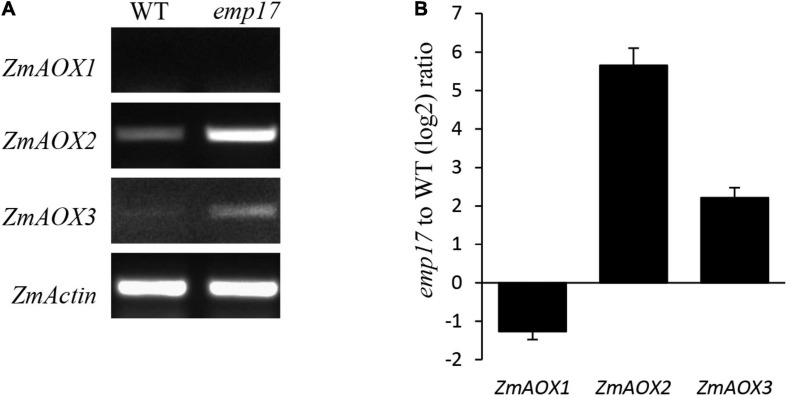
The expression analysis of the *ZmAOX* genes in wild type and *emp17*. **(A,B)** RT-PCR and qRT-PCR were used for analyzing the expression of the *ZmAOX* genes in wild type and *emp17*, respectively. RNA was extracted from 14 DAP embryos and endosperms. qRT-PCR values represent three biological replicates and are normalized against *ZmActin*. Error bars represent the ± SD.

As previously reported, blocking of the cytochrome pathway often induces the alternative non-phosphorylating pathway in the respiratory chain (Yang et al., 2017; Wang et al., 2019; Xu et al., 2020). The maize genome hosts three *ZmAOX* genes (*ZmAOX1, ZmAOX2*, and *ZmAOX3*) (Karpova et al., 2002). As indicated by RT-PCR and qRT-PCR results, the level of *ZmAOX2* and *ZmAOX3* transcripts was dramatically increased in *emp17*, especially *ZmAOX2* (Figures 7A,B). Western blotting analysis revealed that *ZmAOX* is expressed at low levels in the wild type kernels, but drastically enhanced in *emp17* (Figure 6F).

To assess the physiological impact, we then measured the *V*_*t*_, *V*_*alt*_, and *V*_*cyt*_ by using a Chlorolab II liquid-phase oxygen electrode, and specific inhibitors SHAM and KCN, respectively (Wang et al., 2015). The ratios of *V*_*alt*_ to *V*_*t*_ and *V*_*cyt*_ to *V*_*t*_ in *emp17* and wild type kernels were calculated. The results indicated that *V*_*t*_ and *V*_*cyt*_ in *emp17* were decreased to about 18 and 10% in comparison with that in wild type, respectively (Table 1). The ratio of *V*_*alt*_/*V*_*t*_ (75%) in *emp17* is higher than that by about twofold (43%) in wild type. These data confirm that mitochondrial complex III dysfunction severely reduces the cytochrome respiration and induces the alternative respiration pathway in *emp17*.

**TABLE 1:** The respiration rate is altered in WT and *emp17*.

	Respiration rate (nmol O_2_ min^–1^ g^–1^ fresh weight)				
	*V*_*t*_	*V*_*alt*_	*V*_*cyt*_	*V*_*alt*_/*V*_*t*_ (%)	*V*_*cyt*_/*V*_*t*_ (%)
WT	482.49 ± 15.41	208.38 ± 13.91	431.11 ± 17.02	43.19	89.35
*emp17*	88.57 ± 2.99^∗^	66.52 ± 2.67^∗^	45.89 ± 2.33^∗^	75.1	51.81

Mitochondrial *V*_*t*_, *V*_*cyt*_, and *V*_*alt*_ were measured by theChlorolab II liquid-phase oxygen electrode. The activity of cytochrome *c* oxidase and an alternative oxidase were inhibited by 2 mM KCN and 2 mM SHAM, respectively. Data are mean values SEs from three independent biological samples. Statistical differences (*P* < 0.01*) in the same row were based on student’s *t*-test analysis.

*V_*t*_, total respiration rate; *V*_*cyt*_, cytochrome pathway capacity; *V*_*alt*_, alternative pathway capacity; KCN, potassium cyanide; SHAM, salicylhydroxamic acid; SEs, standard errors.*

### Co-evolution Between EMP17 and Editing Sites *ccmF*_*C*_-799 and *nad2*-677

Putative orthologous proteins of EMP17 can be found in many sequenced plant species in the NCBI and Uniport database (Figure 1B). Phylogenetic analysis revealed a high degree of conservation of putative EMP17 orthologs in *A*. *trichopoda* and mono- and dicotyledonous species. EMP17 shared an over 50% sequence identity with most of its orthologous proteins (Figure 1B), i.e., 54.7% with that in *A*. *trichopoda*, the single living species of the sister lineage to all other extant flowering plants (Soltis et al., 2011). However, a putative EMP17 ortholog cannot be found in *O*. *sativa, A*. *thaliana, B*., and *G*. *hirsutum*. The most closely related protein in rice is Os12g0109300, sharing a 40.99% sequence identity with EMP17. In turn, Os12g0109300 shares an 81.94% sequence identity with maize protein GRMZM5G811022. As such, Os12g0109300 is unlikely to be an EMP17 ortholog in rice. The most closely related homologs of EMP17 are OTP82 in *A*. *thaliana*, hypothetical protein XP_013641116 in *B*. *napus* (accession number XP_013641116), and hypothetical protein (accession number KAG4215874) in *G*. *hirsutum*. All of these proteins share a less than 38% sequence identity with EMP17, much lower than that with the *A*. *trichopoda* homolog (54.7%). The results suggest that these species may have lost the *Emp17* gene in the genome.

This raises the question of why this protein is conserved in some species, but lost in others. The gDNA sequence of CcmF_C_ shows that the *ccmF_*C*_-*799 site is “T” in *A*. *trichopoda* and eudicots. However, in monocots, both “T” and “C” present (Supplementary Figure 5A). In all the species harboring *ccmF_*C*_-*799C in mitochondrial DNA, putative orthologs of EMP17 can be identified in the nuclear genomes (*Phoenix dactylifera, Z*. *mays, Sorghum bicolor*, and *T*. *aestivum*) (Supplementary Figure 5A). In the species with *ccmF_*C*_-*799T in the mitochondrial gene, putative ortholog of EMP17 may be lost (Supplementary Figure 5A). A consistent result can be found between the existence of EMP17 orthologs in the nuclear genome and the *nad2*-677 site in mitochondria as well (Supplementary Figure 5B). These results suggest that EMP17 orthologs probably exist in the early flowering plants that do not require the editing function of EMP17 at the *ccmF*_*C*_-799 or *nad2*-677 sites because both sites are “T.” But later in evolution, when the “T” was mutated to “C,” EMP17 was recruited for the editing function. For the species that maintain a “T” at this site, the *Emp17* orthologs may be degenerated or lost. This notion offers a possible explanation for the disappearance of a clear EMP17 ortholog in *O*. *sativa, A*. *thaliana, B*. *napus, G*. *hirsutum*, and possibly in other species as well.

## Discussion

### EMP17 Functions in the Editing at *ccmF*_*C*_-799 and *nad2*-677 and Is Essential for Seed Development in Maize

This study provides strong evidence that EMP17, a previously uncharacterized PPR-DYW protein, functions in the editing of mitochondrial transcripts in maize. Since only 6 out of the 82 maize PPR-DYW proteins have been fully characterized so far, the elucidation of the EMP17 function adds a new piece of information to the repertoire of this large protein family. Our results show that EMP17 is exclusively localized in mitochondria (Figure 1C), and loss-of-function in EMP17 abolishes the editing at *ccmF*_*C*_-799 and *nad2*-677 sites while reducing the editing at *ccmF*_*C*_-906 and *ccmF*_*C*_ -966 sites (Figure 4A). Conversely, the expression of *Emp17* restores the editing defects in the *emp17* mutant (Figure 4A), demonstrating that EMP17 is required for the editing at these sites. In addition, based on the “one PPR motif: one nucleotide” recognition codes (Barkan et al., 2012), the 6,1’-amino acid residue combinations of EMP17 align well with the upstream sequences of *ccmF*_*C*_-799 and *nad2*-677 sites, and weakly with those of the *ccmF*_*C*_-906 and -966 sites (Figure 4B), suggesting that EMP17 may recognize its substrates specifically. Furthermore, the lack of editing is accompanied by a reduced mitochondrial complex III assembly, inhibition of the cytochrome pathway, elevated alternative pathway, and severely reduced respiration rates in the *emp17* mutants. All of these results provide convincing evidence that EMP17 functions on the C-to-U editing at these sites in mitochondria and the loss of function of EMP17 impairs the cytochrome respiratory pathway.

The severely reduced assembly of complex III suggests that the impaired OXPHOS chain can likely be owed to the loss of editing at the *ccmF*_*C*_ sites in *emp17* (Figure 4A). Except for the three *ccmF*_*C*_ sites and the *nad2*-677 site, no other defects were found in the transcripts that are directly associated with complex III. In the four mitochondrion-encoded CCM pathway proteins (CcmB, CcmC, CcmF_N_, and CcmF_C_) that are essential for the CCM and biogenesis of complex III (Giege et al., 2008), no defects were detected in the transcripts of *ccmB, ccmC*, and *ccmF*_*N*_. Additionally, expression levels of *ccmB, ccmF*_*C*_, and *ccmF*_*N*_ in *emp17* were either indistinguishable from that in wild type, or increased (*ccmC*) (Supplementary Figure 4). CcmF_C_ has been shown to be important for plant growth and development, as the loss of *ccmF*_*C*_ expression led to a deficiency of the *c*-type cytochromes and complex III in *wtf9* (Francs-Small et al., 2012). Consistent with that, the Cyt*c*_1_ and Cyt*c* proteins were barely detectable (Figure 6F), and the assembly of complex III was severely decreased in *emp17* (Figure 6C). These results suggest that the unedited *ccmF*_*C*_-799 disrupts the complex III assembly and leads to a dysfunction of CCM.

The editing deficiency at the *ccmF*_*C*_-799 site causing the Ser^267^-to-Pro^267^ change in CcmF_C_ is probably the major cause for the inhibited kernel development in *emp17*. In plant mitochondria, Cyt*c* and Cyt*c*_1_ are the essential components of mitochondrial complex III in the OXPHOS chain. Maturation of Cyt*c* and Cyt*c*_1_ is crucial to mitochondrial functions, and hence, to plant growth and development. Impairment of *c*-type cytochrome maturation arrests seed development or plant growth. A deficiency of mature Cyt*c*_1_ and Cyt*c* in the *ccmh* mutant causes embryo lethality in *Arabidopsis* (Meyer et al., 2005). Similarly, a deficiency of Cyt*c*_1_ and Cyt*c* resulting from abolished editing at the *ccmF*_*N*_-1553 and -1357 sites in *emp7* and *ppr27*, respectively, results in embryo lethality in maize (Sun F. et al., 2015; Liu et al., 2020). The lack of Cyt*c*_1_ and Cyt*c* blocks the assembly of mitochondrial complex III, decreases the cytochrome respiration rate, and elevates the alternative non-phosphorylating pathway in the *emp7* and *ppr27* mutants. For the *emp17* mutants, we found consistent results. The assembly of complex III was severely inhibited in *emp17*, and *V*_*t*_ and *V*_*cyt*_ were decreased to about 18 and 10% in comparison with that in wild type, respectively (Table 1), and the expression of *ZmAOX2* and *ZmAOX3* was dramatically increased in *emp17* compared with wild type (Figures 6F, 7A,B). Thus, the lack of Cyt*c*_1_ and Cyt*c* and severely reduced biogenesis of mitochondrial complex III blocks the cytochrome pathway and impairs the kernel development.

Our data suggest that the abolished editing at the *nad2*-677 site is probably not a major cause for the defective seed development in *emp17*. The editing deficiency at *nad2*-677 results in a Phe-to-Ser change at Nad2-226. As an essential component of the mitochondrial complex I, a deficiency of Nad2 impairs the assembly of complex I and arrests kernel development in maize (Xiu et al., 2016; Yang et al., 2020). However, the Phe-to-Ser change at Nad2-226 in *emp17* does not significantly affect the assembly of mitochondrial complex I, or its activity as determined by in-gel NADH dehydrogenase activity (Figure 6B). Although we cannot rule out the possibility that the detected assembled complex I is in fact non-functional in the electron transfer chain, it is likely that the amino acid residue change in Nad2-226 may have a less detrimental impact on the Nad2 function.

### The Ser^267^ Residue in CcmF_C_ Is Essential for the CcmF_C_ Function

**FIGURE 8:**
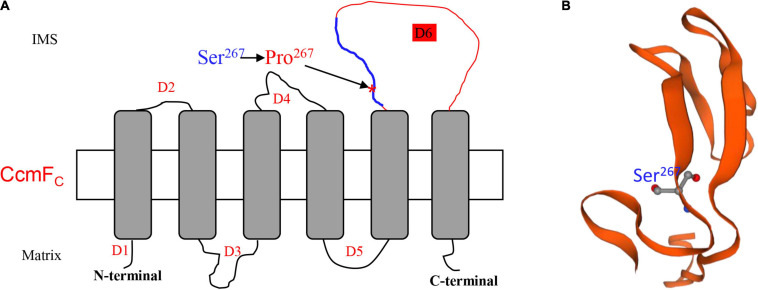
Ser^267^ is in the third intermembrane space loop named Domain 6 (D6). **(A)** Topology model of ZmCcmF_C_ predicted by TMHMM Server v. 2.0 (http://www.cbs.dtu.dk/services/TMHMM/). The Ser^267^ was marked by arrow. D1-6, Domain1-6; IMS, Intermembrane space. **(B)** The predicted protein structure of peptide from 258th to 302th in ZmCcmF_C_ marked by blue line in **(A)**.

The lack of editing at *ccmF*_*C*_-799 as a result of the *Emp17* mutation constitutes a surrogate mutation of Ser^267^ to Pro^267^ in the CcmF_C_ protein. The severe impact of this mutation on CCM and complex III assembly illustrates the importance of the Ser residue at CcmFc-267 for the CcmF_C_ function. In bacteria and plant mitochondria, CcmF and CcmH are proposed to take part in the final step of CCM, ligating heme delivered by CcmE to apo-cyt*c* (Giege et al., 2008; Rayapuram et al., 2008). In plant mitochondria, the *ccmF* gene has been split into multiple genes. For instance, *ccmF* is split into three genes (*ccmF_*N*1_, ccmF_*N*2_*, and *ccmF*_*C*_) in *Arabidopsis* (Unseld et al., 1997), and two genes (*ccmF*_*N*_ and *ccmF*_*C*_) in maize (Clifton et al., 2004). Structure prediction and trypsin digestion experiments suggest that AtCcmF_C_ has six transmembrane helices, three intermembrane space loops, and four mitochondrial matrix domains (Rayapuram et al., 2008). Transmembrane helice prediction by the TMHMM Server v. 2.0^3^ indicates that the structure of ZmCcmF_C_ and AtCcmF_C_ is quite similar. The two proteins share a 78% sequence identity. Based on this structure, Ser^267^ is located in the third intermembrane space loop named domain 6 (D6) of CcmF_C_ (Figure 8A). Alignment of the protein sequences shows that the Ser^267^ in various CcmF_C_ proteins is highly conserved across species (Figure 5A), suggesting its importance to CcmF_C_ function. It is known that Pro residue is a disruptor of protein α-helix and not favored in the β-sheet structures, as it has structure is limited and cannot complete the H-bonding network. For example, the Leu-to-Pro change in Nad7-279 and Atp6-213 and in the α-helix region is attributed to the destruction of the Nad7 and Atp6 function in the maize mutant *emp18* and *smk1* (Li et al., 2014, 2019). The Ser^267^ in CcmF_C_ was predicted in a β-sheet by the Swiss-model algorithm^4^ (Figure 8B). Proline is not favored in β-sheet structures as it cannot complete the H-bonding network. It is possible that the Ser^267^-to-Pro^267^ mutation in CcmF_C_ in the β-sheet may negatively impact the structural stability of D6 that leads to non-functional CcmF_C_. The exact function of the D6 intermembrane space loop in CcmF_C_ is unknown, however, these results imply that the Ser^267^ residue in D6 plays a critical role for the function of CcmF_C_.

## Data Availability Statement

The original contributions presented in the study are included in the article/Supplementary Material, further inquiries can be directed to the corresponding author/s.

## Author Contributions

YW and B-CT designed the research and analyzed the data. YW and X-YL conducted most of the experiments. FS performed the BN gel assay. Z-QH, Y-YL, and Z-QG participated in the linkage and genetic complementarity analysis. XW performed the respiration rate assay. YW, X-YL, Y-ZY, AS, and B-CT wrote the article. All authors contributed to the article and approved the submitted version.

## Conflict of Interest

The authors declare that the research was conducted in the absence of any commercial or financial relationships that could be construed as a potential conflict of interest.

## Publisher’s Note

All claims expressed in this article are solely those of the authors and do not necessarily represent those of their affiliated organizations, or those of the publisher, the editors and the reviewers. Any product that may be evaluated in this article, or claim that may be made by its manufacturer, is not guaranteed or endorsed by the publisher.

## Footnotes

1
http://www.cbs.dtu.dk/services/TargetP

2
http://urgi.versailles.inra.fr/predotar/predotar.html

3
http://www.cbs.dtu.dk/services/TMHMM/

4
https://swissmodel.expasy.org/interactive

## Supplementary Material

Supplementary Figure 1 The alignment of the DYW domains within PpPPR56, PpPPR65, and EMP17.

Supplementary Figure 2 The transcription profiling of *Emp17* in wild type.

Supplementary Figure 3 The Linkage analysis of *emp17*.

Supplementary Figure 4 Transcription profiling of the 35 mitochondrial protein-coding genes in wild type and *emp17*.

Supplementary Figure 5 The co-evolutionary relationship between EMP17 and the editing sites controlled by EMP17.

Supplementary Table 1 Primers used for the study.
