# Supplementary material for: PPR-DYW Protein EMP17 Is Required for Mitochondrial RNA Editing, Complex III Biogenesis, and Seed Development in Maize
Source: Front Plant Sci. 2021 Jul 28;12:693272. doi: 10.3389/fpls.2021.693272 (PMC8357149; doi:10.3389/fpls.2021.693272)
Supplement: Supplementary Figure 1 — The alignment of the DYW domains within PpPPR56, PpPPR65, and EMP17. [file Data_Sheet_1.PDF]

Figure S1

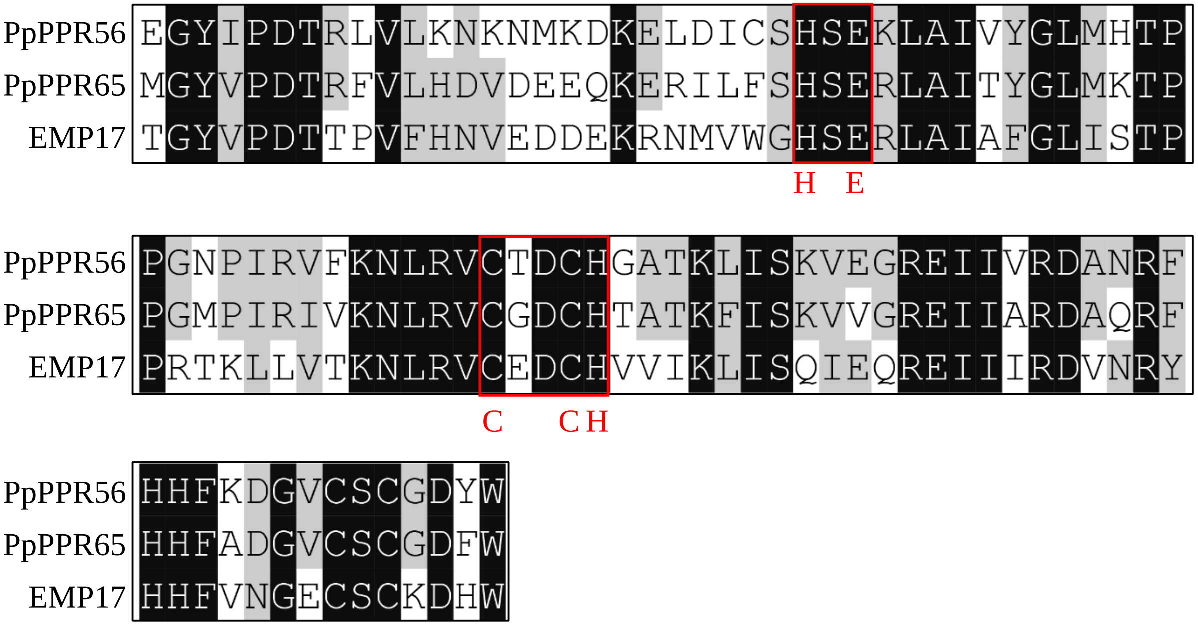

Fig. S1 The alignment of the DYW domains within PpPPR56, PpPPR65 and EMP17.

The conserved cytidine deaminase-like zinc binding signature residues HxE(x)nCxxC are shown in red font.

Figure S2

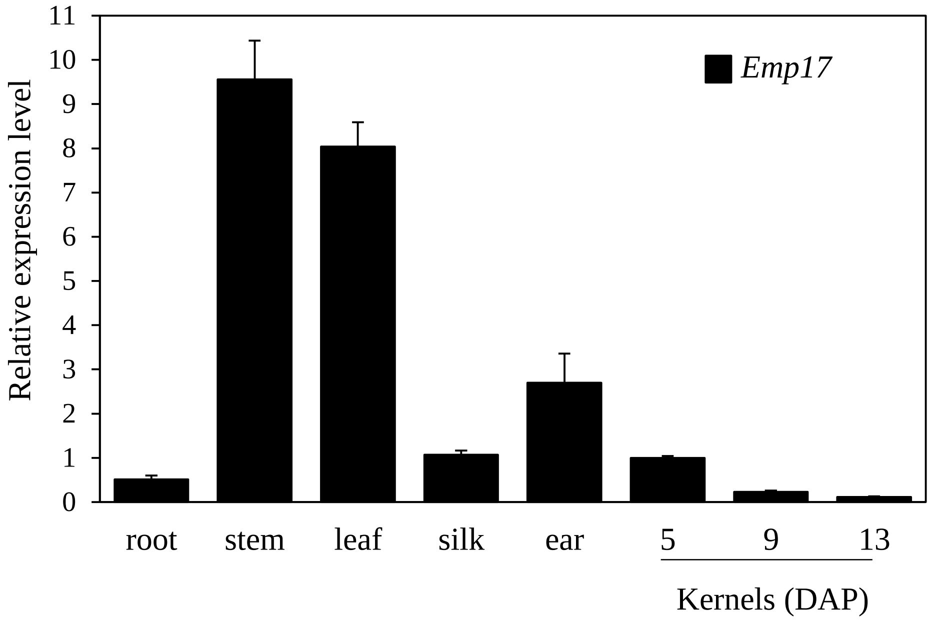

Fig. S2 The transcription profiling of *Emp17* in wild type.

The expression pattern of *Emp17*. Values shown are calculated from the mean of three biological replicates, and the error bars represent the ±SD.

Figure S3

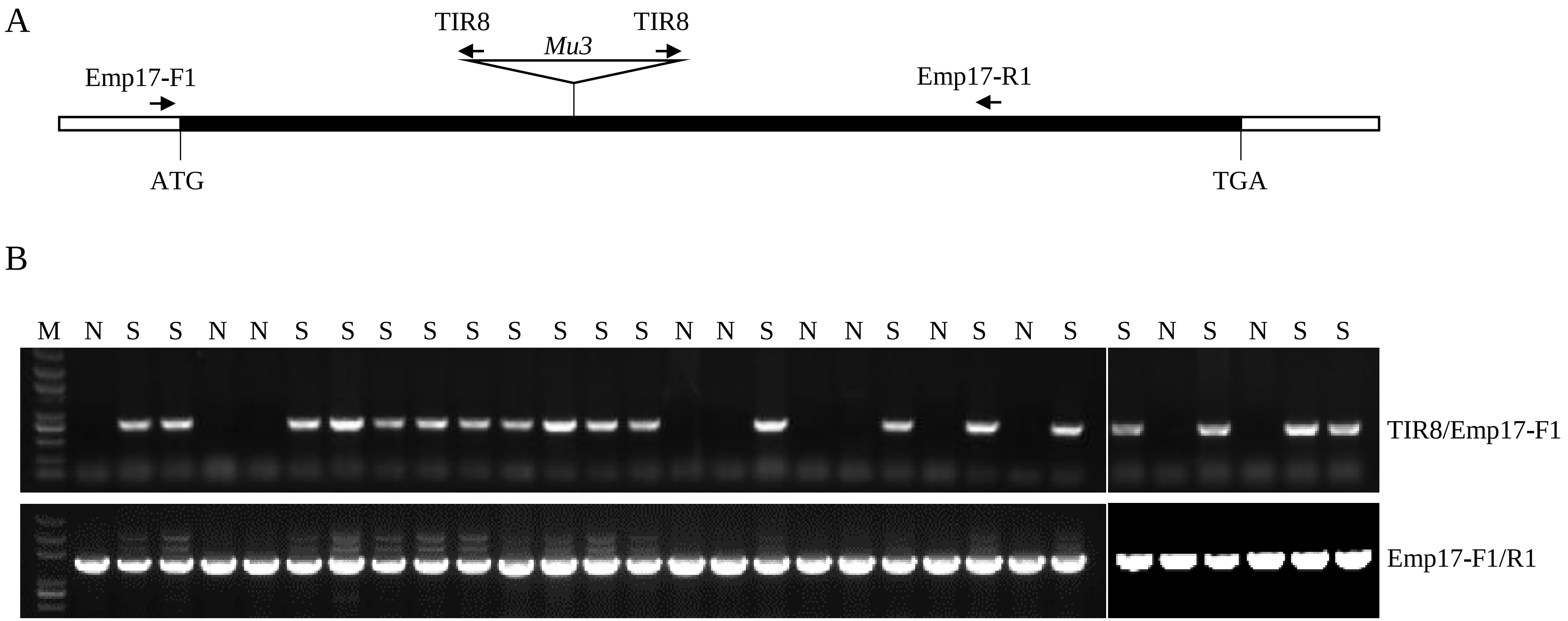

Fig. S3 The Linkage analysis of *emp17*.

(A) The gene structure of *Emp17*. The *Mu* insertion was marked by triangle. The primers (TIR8 and Emp17-F1/R1) were used for genotyping. (B) The F2 population segregating from an *emp17* heterozygous plant. The bands amplified by PCR using TIR8/Emp17-F1 primers indicate the *Mu* insertion in *Emp17*. The bands amplified by PCR using Emp17-F1/R1 primers indicate wild type *Emp17* gene. N, non-segregating; S, segregating.

Figure S4

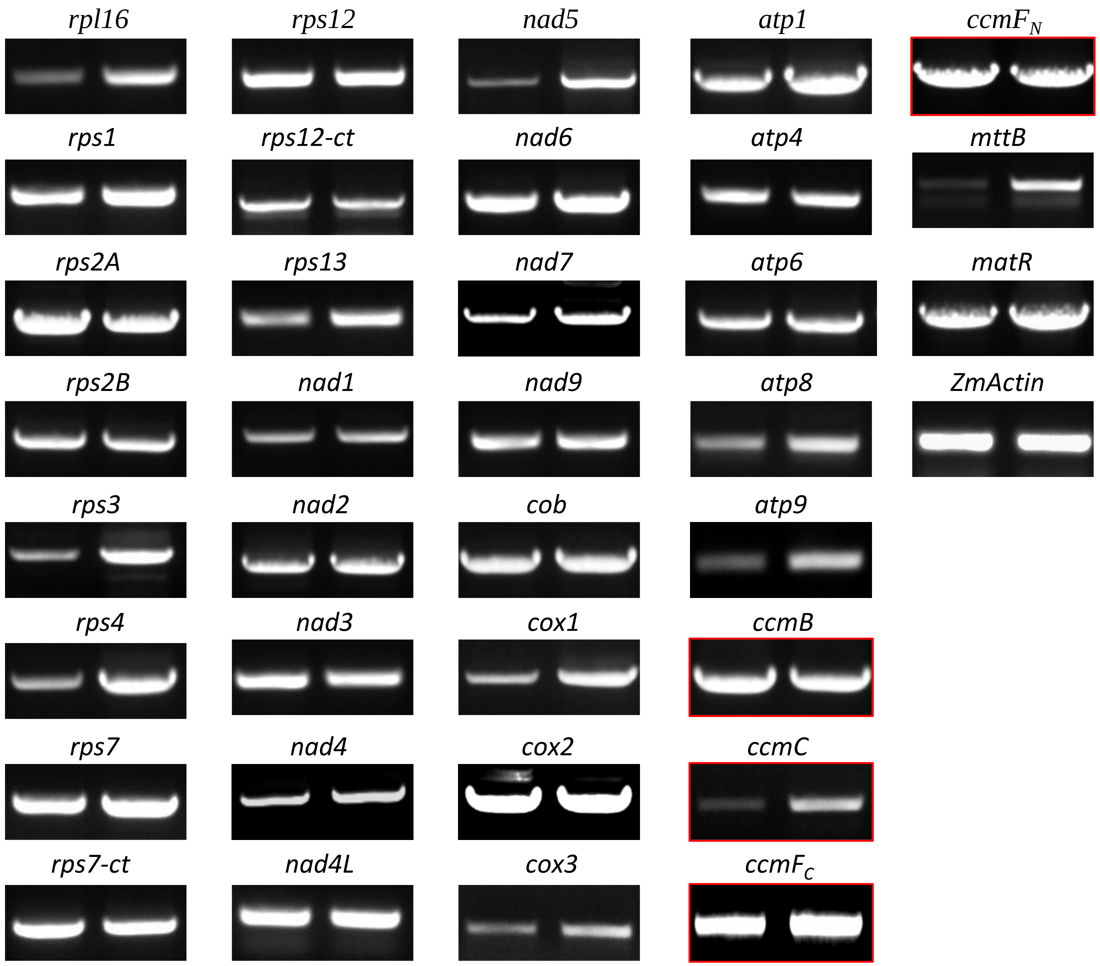

Fig. S4 Transcription profiling of the 35 mitochondrial protein-coding genes in wild type and *emp17*. RNA was extracted from 14 DAP embryos and endosperms. Left: WT, Right: *emp17*. The templates were normalized against *ZmActin*.

Figure S5

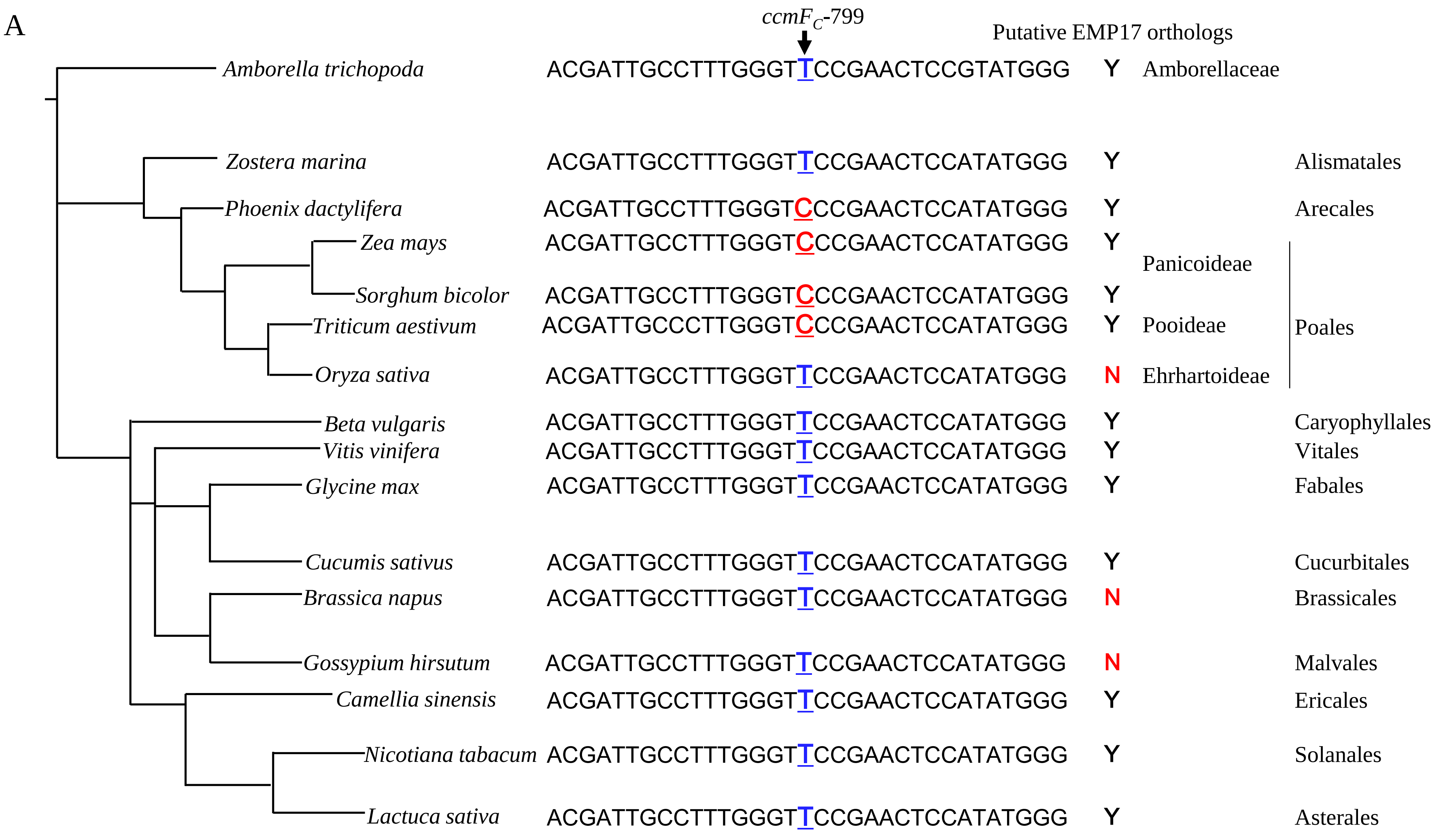

A
ccmFC-799
Putative EMP17 orthologs
Amborella trichopoda ACGATTGCCTTTGGGTTCCGAACTCCGTATGGG Y Amborellaceae
Zostera marina ACGATTGCCTTTGGGTTCCGAACTCCATATGGG Y Alismatales
Phoenix dactylifera ACGATTGCCTTTGGGTCCCGAACTCCATATGGG Y Arecales
Zea mays ACGATTGCCTTTGGGTCCCGAACTCCATATGGG Y
Panicoideae
Sorghum bicolor ACGATTGCCTTTGGGTCCCGAACTCCATATGGG Y
Triticum aestivum ACGATTGCCCTTGGGTCCCGAACTCCATATGGG Y Pooideae
Poales
Oryza sativa ACGATTGCCTTTGGGTTCCGAACTCCATATGGG N Ehrhartoideae
Beta vulgaris ACGATTGCCTTTGGGTTCCGAACTCCATATGGG Y Caryophyllales
Vitis vinifera ACGATTGCCTTTGGGTTCCGAACTCCATATGGG Y Vitales
Glycine max ACGATTGCCTTTGGGTTCCGAACTCCATATGGG Y Fabales
Cucumis sativus ACGATTGCCTTTGGGTTCCGAACTCCATATGGG Y Cucurbitales
Brassica napus ACGATTGCCTTTGGGTTCCGAACTCCATATGGG N Brassicales
Gossypium hirsutum ACGATTGCCTTTGGGTTCCGAACTCCATATGGG N Malvales
Camellia sinensis ACGATTGCCTTTGGGTTCCGAACTCCATATGGG Y Ericales
Nicotiana tabacum ACGATTGCCTTTGGGTTCCGAACTCCATATGGG Y Solanales
Lactuca sativa ACGATTGCCTTTGGGTTCCGAACTCCATATGGG Y Asterales

Figure S5

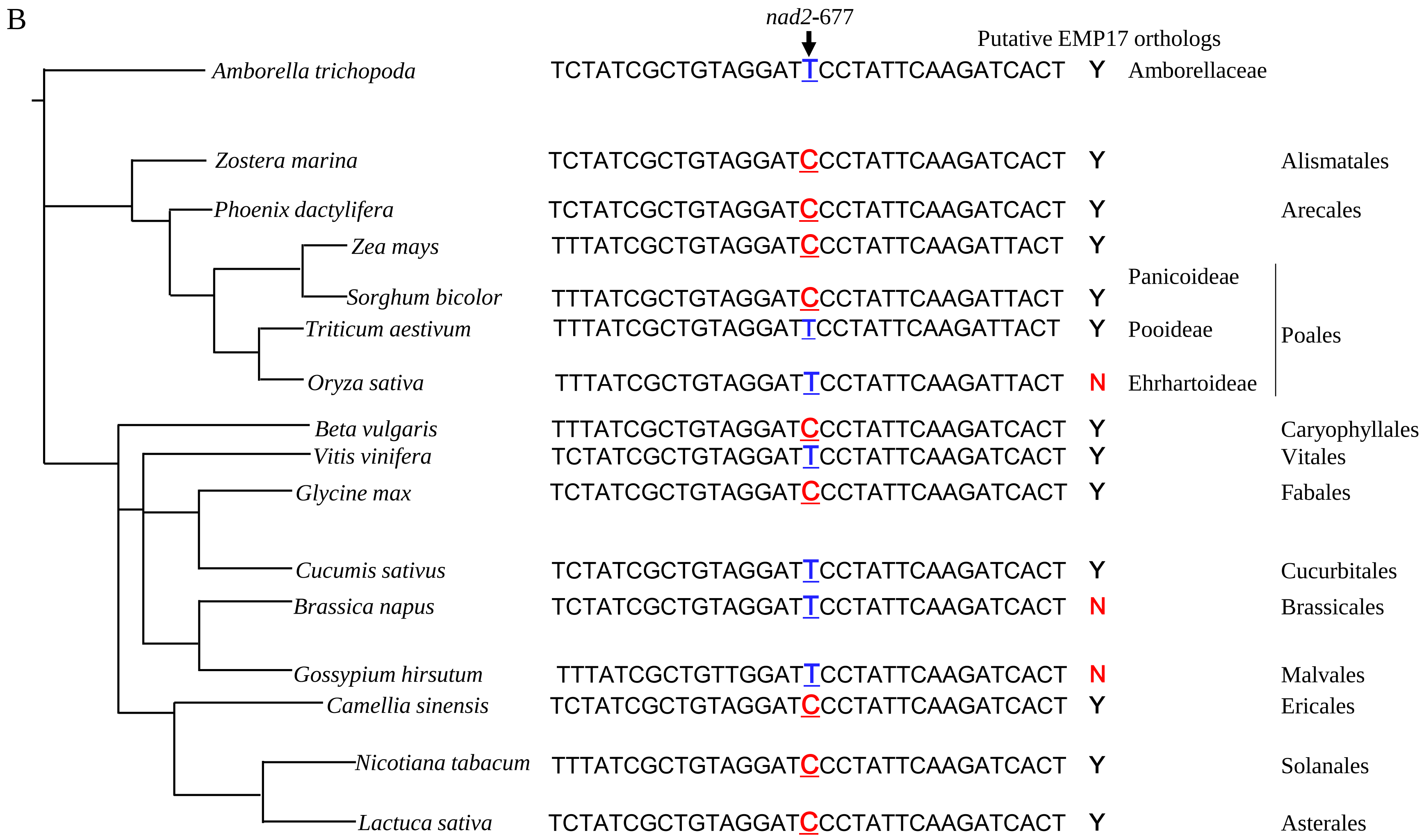

Fig. S5 The co-evolutionary relationship between EMP17 and the editing sites controlled by EMP17.

(A) The co-evolutionary relationship between EMP17 and *ccmF*$_C$-799 site. (B) The co-evolutionary relationship between EMP17 and *nad2*-677 site. The editing sites marked by black arrows. Y: Putative EMP17 orthologs are present in these species; N: No clearly EMP17 ortholog was found in these species.
